# Mistletoes could moderate drought impacts on birds, but are themselves susceptible to drought-induced dieback

**DOI:** 10.1098/rspb.2022.0358

**Published:** 2022-07-13

**Authors:** Ross Crates, David M. Watson, Gregory F. Albery, Timothée Bonnet, Liam Murphy, Laura Rayner, Dejan Stojanovic, Chris Timewell, Beau Meney, Mick Roderick, Dean Ingwersen, Robert Heinsohn

**Affiliations:** ^1^ Fenner School of Environment and Society, Australian National University, Linnaeus Way, Acton, Canberra 2601, Australia; ^2^ Research School of Biology, Australian National University, Linnaeus Way, Acton, Canberra 2601, Australia; ^3^ School of Agricultural, Environmental and Veterinary Sciences, Charles Sturt University, Albury, New South Wales, Australia; ^4^ Department of Biology, Georgetown University, Washington, DC, USA; ^5^ BirdLife Australia, Carlton, Melbourne, Australia

**Keywords:** climate change, population monitoring, ecosystem resillience, food webs, global change, phenology

## Abstract

Mistletoes are hemiparasitic plants and keystone species in many ecosystems globally. Given predicted increases in drought frequency and intensity, mistletoes may be crucial for moderating drought impacts on community structure. Dependent on host vascular flows, mistletoes can succumb to stress when water availability falls, making them susceptible to mortality during drought. We counted mistletoe across greater than 350 000 km^2^ of southeastern Australia and conducted standardized bird surveys between 2016 and 2021, spanning a major drought event in 2018–2019. We aimed to identify predictors of mistletoe abundance and mortality and determine whether mistletoes might moderate drought impacts on woodland birds. Live mistletoe abundance varied with tree species composition, land use and presence of mistletoebirds. Mistletoe mortality was widespread, consistent with high 2018/2019 summer temperatures, low 2019/2020 summer rainfall and the interaction between summer temperatures and rainfall in 2019/2020. The positive association between surviving mistletoes and woodland birds was greatest in the peak drought breeding seasons of 2018/2019 and 2019/2020, particularly for small residents and insectivores. Paradoxically, mistletoes could moderate drought impacts on birds, but are themselves vulnerable to drought-induced mortality. An improved understanding of the drivers and dynamics of mistletoe mortality is needed to address potential cascading trophic impacts associated with mistletoe die-off.

## 1. Introduction

Mistletoes—five families of flowering plants in the order Santalales—are hemiparasites with over 1600 species distributed globally [1]. Given their well-documented ecological roles in nutrient cycling, forest stand dynamics, food and nest substrate provisioning, mistletoes are keystone species in many ecosystems [2]. In addition to providing abundant resources and boosting heterogeneity in productivity via nutrient subsidies, these plants depend on a network of other organisms, including pollinators, seed dispersers and host plants. Consequently, mistletoe health and abundance serve as important bioindicators of broader ecosystem health [3–5].

Although population-scale impacts of climate change on mistletoes have not been quantified, several aspects of their life history and physiology pre-dispose them to acute sensitivity to sudden changes in water availability. Lacking roots and storage organs, mistletoes use high transpiration rates to maintain vascular flow from hosts [6,7]. By retaining cations in semi-succulent foliage, mistletoes maintain water balance by passively drawing down a concentration gradient [8,9]. While this enriches tissues, increases water flux and likely within-canopy humidity [10], their limited control over stomatal closure makes mistletoes sensitive to sudden reductions in moisture availability [7], with increased evapotranspiration from prolonged hot/dry conditions associated with mistletoe mortality [11,12].

In Australia, flowering mistletoes provide high-quality nutritional resources for many animals [13]. In addition to being the principal food source for several nectarivorous bird species [14,15]*,* many other species rely on mistletoe fruits for carbohydrates, fats, amino acids and water [2,16]. Annual flowering and fruiting phenology of mistletoes is typically more regular than that of their host trees [13]. Sympatric mistletoe species often exhibit complementary periods of peak flowering and fruiting, thereby extending the period of nectar and fruit availability in a given location [17]. Consequently, mistletoes provide predictable and reliable resources during droughts when ecosystem productivity such as eucalypt flowering is otherwise low [16]. Lush, dense foliage of healthy mistletoes are a key browsing resource for arboreal mammals and create microclimates that moderate temperature extremes [10,18], making mistletoe favourable nesting and roosting sites for many bird species [9]. With predicted increases in the frequencies of prolonged droughts and severe heatwaves under climate change [19,20], more species may depend on live mistletoe to survive such events in coming decades.

There is some evidence that mistletoe mortality events have occurred in southeastern Australia in recent years [21,22]. Potential drivers of mistletoe mortality in Australia's woodlands include the 2019/2020 megafires [23] and drought-induced eucalypt dieback associated with the 2018/2019 drought event [24,25]. Three widespread species potentially affected by drought are box *Amyema miquelii*, long-flowered *Dendrophthoe vitellina* and needle-leaf mistletoe *Amyema cambageii.* These species provide key breeding resources for many threatened species including the Critically Endangered regent honeyeater *Anthochaera phrygia* [26,27]. In particular, needle-leaf mistletoes provide nectar resources in riparian zones that function as important drought refugia [28]. If mistletoe mortality is widespread, it could have knock-on impacts across food webs, interrupting nutrient returns, microclimatic buffering and food availability [2]. However, current monitoring data available to quantify the causes, extent and potential impact of mistletoe mortality in woodland ecosystems are limited in extent, hindering current capacity to address threats through conservation actions.

Here we address some current shortfalls in our knowledge of the predictors of mistletoe abundance, mistletoe mortality and the importance of mistletoe in sustaining biodiversity at higher trophic levels during climate extremes. We generated a baseline dataset to monitor long-term mistletoe population dynamics and associated woodland bird abundance. With these data, we aimed to answer two questions:

Question 1: what are the predictors of mistletoe abundance and the drivers of mistletoe mortality?
Question 2: what is the relationship between live mistletoe abundance and woodland bird abundance, and how does this relationship change during drought?

## 2. Methods

### (a) Habitat assessments and mistletoe counts

**Figure 1.**
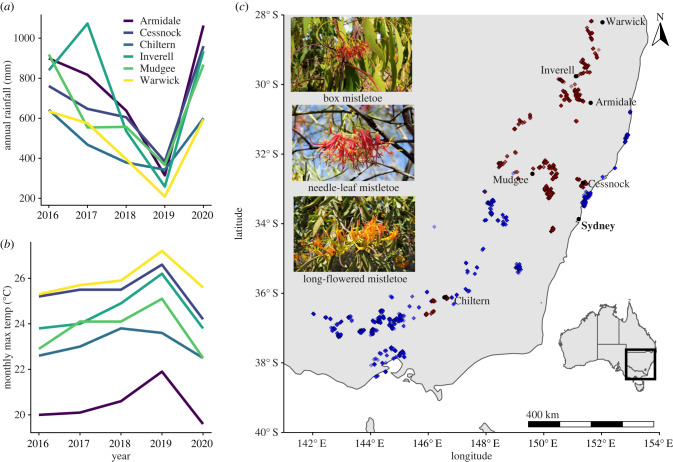
(*a*,*b*) Annual rainfall and mean monthly maximum temperature data for weather stations spanning the spatial and temporal extent of the bird monitoring dataset. (*c*) Distribution of both woodland bird and mistletoe (red) and mistletoe only (light blue) monitoring sites in southeastern Australia. Top left inset: box *Amyema miquelii*, long-flowered *Dendrophthoe vitellina* and needle-leaf *Amyema cambageii* mistletoe species included in the study. Bottom right inset: study range on a national scale. Place labels (excluding Sydney) show the location of the summary climate data presented in (*a*,*b*). Rainfall and temperature data are shown to summarize annual variation in the climate surface data used in mistletoe models, sourced from the Australian Bureau of Meteorology http://www.bom.gov.au/climate/data/, accessed 9/3/2021. (Online version in colour.)

During 2019/2020, we conducted habitat assessments at 2111 monitoring sites spanning over 300 000 km^2^ of southeastern Australia (figure 1). We selected monitoring sites in areas of woodland habitat deemed suitable for two Critically Endangered bird species: the regent honeyeater and swift parrot *Lathamus discolor.* We used a combination of MaxEnt habitat suitability models (electronic supplementary material, figures S1 and S2), expert field searches and the location of previous sightings to inform site locations. Since both bird species are habitat specialists [29], our sampling encompasses the highest quality remaining woodlands in southeastern Australia.

Each monitoring site was a 50 m radius (0.79 hectares) around a fixed GPS location to 2 m accuracy. During one visit to each site in 2019/2020, we recorded the habitat fixed effects detailed in table 1. Our mistletoe counts focussed on the three most abundant mistletoe species (family Loranthaceae) in the study range: box, needle-leaf and long-flowered*.* We conducted a 360° search of the canopy from each site centroid, deviating from this point where necessary to count accurately the number of live and dead mistletoe clumps present.

**Table 1.** Site-level and visit-level fixed effects obtained for identifying predictors of mistletoe abundance and health and to model the effect of mistletoe abundance on woodland bird abundance. For further information on the fixed effects, see electronic supplementary material, table S1.

level	fixed effect	description
site-level	spatial location	WGS84 decimal latitude longitude to 2 m accuracy
	region	10-level factor defining regional clusters of monitoring sites. Included as a random term in mistletoe and bird models
	land use	9-level factor: primary land use
	canopy cover	percentage canopy cover to the nearest 5%
	tree species PC1 and PC2	principal component axes 1 and 2 of tree species composition (see electronic supplementary material, figure S3)
	tree age	proportion of trees present with a diameter at breast height >80 cm
	tree health	proportion of trees in the site that are healthy or only mildly stressed per Briggs & Taws [30]
	shrub cover	percentage shrub cover (vegetation 30 cm to 2 m) to the nearest 5%
	live mistletoe	total number of clumps of live mistletoe across all three species
	dead mistletoe	total number of clumps of dead mistletoe across all three species
	distance to permanent or semi-permanent water source	5-level factor: 1 = water present within site, 2 = water within 100 m, 3 = water within 300 m, 4 = water >300 m away, 5 = distance to water unknown
	mistletoebird presence	presence/absence of mistletoebirds detected during ≥ 1 bird survey per site
	noisy miner abundance	mean abundance of noisy miners (a hyperabundant and colonial native bird that excludes other songbirds from habitats they occupy) detected during repeat bird surveys at each site (mistletoe models), or abundance per site visit (bird models)
visit-level	breeding season	annual Austral breeding season August to January
	hours since dawn/to dusk	hours from 06.00 (morning) or hours to 19.00 (afternoon)
	observer	7-level factor: bird surveyor/habitat assessor. Random effect in bird models
	blossom	5-level factor: site-level blossom abundance (including both eucalypts and mistletoes): 0 = no blossom; 1 = light blossom: few flowers in a small number of trees; 2 = moderate blossom: few flowers in many trees or moderate flowering in a few trees; 3 = heavy blossom: profuse flowering in few trees or moderate flowering in multiple trees; 4 = very heavy blossom: multiple profusely flowering canopies
	max summer temperature	mean monthly maximum summer temperature November to February
	max summer rain	mean maximum monthly summer rainfall November to February

### (b) Bird surveys

We conducted 9012 point-count surveys at a total of 1218 monitoring sites in the Austral spring/summer breeding season (August to January) between 2016 and 2021 (electronic supplementary material, table S2). Each survey was conducted by one of 15 professional ornithologists, with 86% of surveys completed by seven observers. Our rapid (5-min) census, involving 1 min of regent honeyeater song broadcast, was designed to maximize the detectability of such rare, nomadic habitat specialists by increasing the spatial extent of surveys without compromising detectability [31]. We recorded the maximum count of all bird species detected visually or aurally within a 50 m radius of the fixed-point location during each site visit, along with a blossom score for each site. Observers remained at the site centroid as much as possible but deviated where necessary to identify individual birds to species level or to obtain accurate counts of birds occupying heavily flowering trees near site boundaries. We did not include transient birds flying through or over study sites in the counts. The blossom score was a five-level factor (table 1); a simple way of modelling variation in blossom abundance on nomadic species occupancy patterns [32]. To account for intra-seasonal variation in flowering phenology and associated changes in woodland bird distribution/abundance [33], we surveyed as many sites as possible (77%) twice; once in spring between August and October and again in early summer between November and January.

### (c) Climate data

We sourced climate data from the Australian National University Climate surface database (ANUCLIM v. 6.1 [34]). We obtained national monthly maximum temperature and rainfall measures between 2017 and 2020 and derived these measures for each of our monitoring site locations from a 250 m national raster. See electronic supplementary material, file S1, for further information on derivation of the climate data. For mistletoe analysis, we calculated annual mean maximum rainfall and temperature measures averaged across the summer months of November to February, when mistletoes are most susceptible to drought impacts [35].

### (d) Statistical analysis

We used R v. 3.4.3 [36] for all statistical analyses. We first checked for spatial autocorrelation in mistletoe abundance and mortality data using correlograms of Moran's I via package *ncf* v. 1.2-5 [37]. To check for cross-correlation between covariates, we used *GGally* v. 1.4.0 [38], but no covariates showed consistent strong positive or negative correlation with others.

To account for interspecific variation in the suitability of tree species as mistletoe hosts [39], we ran a centred and scaled principal component analysis (PCA) of the proportional contribution of each tree species to canopy cover across sites using *stats* v. 3.6.2 in base R. The first two principal component axes, which we included in subsequent mistletoe and bird models (tree species PC1 and PC2), together explained 9% of the total variation in tree species composition. Because the proportion of total variation explained by the PCA was relatively low, we also conducted a separate analysis of the association between individual tree species and live mistletoe abundance (electronic supplementary material, file S2).

To account for spatial autocorrelation in the mistletoe and bird data, we fitted a series of integrated nested Laplace approximation (INLA) generalized linear mixed models (GLMMs) via package *INLA* v. 21.02-23 [40]. The INLA models included a stochastic partial differentiation equation (SPDE) random term that calculates the distances between the spatial location of monitoring sites using Matern covariance [41,42]. We selected the best models as those with the lowest deviance information criterion (DIC) value and assessed their goodness of fit based on conditional *R*^2^-values [43].

To answer question 1, *what are the predictors of mistletoe abundance and the drivers of mistletoe mortality?,* we first used live mistletoe counts as the response in a GLMM with a negative binomial error structure. The model included as fixed effects: land use, canopy cover, tree species composition, tree health, tree age and distance to standing water, with region included as a random term (table 1). To assess the association between mistletoebird presence (the key disperser of mistletoe fruits [15]) and noisy miner abundance (a key driver of mistletoebird distribution [44]) on mistletoe abundance, we re-ran the model on the subset of sites where we conducted bird surveys (figure 1) and included mistletoebird presence and noisy miner abundance as fixed effects in the saturated model.

We replaced live mistletoe abundance with dead mistletoe abundance as the response measure to identify the predictors of mistletoe mortality. To the fixed effects included in the live mistletoe model described above, we added live mistletoe abundance, mean maximum monthly summer (Nov–Feb) temperature and summer rainfall for 2017/2018, 2018/2019 and 2019/2020, as well as the annual interaction between these temperature and rainfall measures.

To answer question 2, *what is the relationship between live mistletoe abundance and woodland bird abundance, and how does this relationship change during drought?,* we calculated four woodland bird abundance response measures, based on overall bird abundance, body size, residency status and feeding guild (table 2 and electronic supplementary material, table S2). We excluded noisy miners from bird abundance measures due to their impacts on woodland bird abundance [44], and instead included noisy miner abundance as a fixed effect in the woodland bird models (table 1). The response measures were counts of birds described in table 2. Fixed effects included overall blossom score, breeding season, noisy miner abundance, canopy and shrub cover extent, tree species composition, land use, distance to standing water, survey time and live mistletoe abundance (log + 1 transformed). We included observer and region as random terms. To examine how the relationship between woodland birds and mistletoes changed during drought, we included in the woodland bird models the interaction term *live mistletoe abundance × breeding season*. We also conducted supplementary analyses to further explore the relative importance of mistletoe and eucalypt blossom during the drought for woodland birds (electronic supplementary material, file S3). For all bird models we used a Poisson error structure.

**Table 2.** Bird response measures used in models to answer question 2: *what is the relationship between live mistletoe abundance and woodland bird abundance, and how does this relationship change during drought?* Note the species composition of bird response measures are not mutually exclusive. See electronic supplementary material, table S3 and the raw dataset available via the Dryad Digital Repository [62].

bird response	description	justification
total bird abundance	total abundance of all bird species detected, excluding noisy miners	overall bird abundance is the ultimate measure of bird community response to mistletoe health and abundance [45]
small resident bird abundance	abundance of all birds with mean body mass less than 60 g considered not to be migratory or nomadic	60 g is the mean mass of noisy miners, which exclude smaller birds from habitats they occupy. Excluding migratory and nomadic species accounts for high spatio-temporal variability of such species, independent of any effects of mistletoe abundance on bird abundance [27,44]
nectarivores	total abundance of all nectarivorous birds	feeding guilds will differ in the extent to which they depend on mistletoe abundance. Nectarivores predicted to be most dependent on mistletoes as a direct feeding substrate [46]
insectivores	total abundance of all insectivorous birds	insectivores predicted to be less dependent on mistletoes than nectarivores, but through potential impacts of mistletoe on insectivore abundance, insectivores may be more dependent on mistletoe than granivores [14,15]

## 3. Results

Live mistletoe was present at 1267 of 2111 sites. Where present, the median number of live clumps per site was 10 (s.d. = 22). Dead mistletoe was present at 1008 sites and where present, the median number of dead mistletoe clumps per site was four (s.d. = 12). The proportion of dead mistletoe was highly variable and substantial in some areas—even where total mistletoe abundance was high (electronic supplementary material, figure S4). There was positive spatial autocorrelation in the proportion of dead mistletoe present at monitoring sites out to 100 km but was greatest as the local scale from 0–6 km (electronic supplementary material, figures S4 and S5).

*Question 1: what are the predictors of live mistletoe abundance and the drivers of mistletoe mortality?*

**Figure 2.**
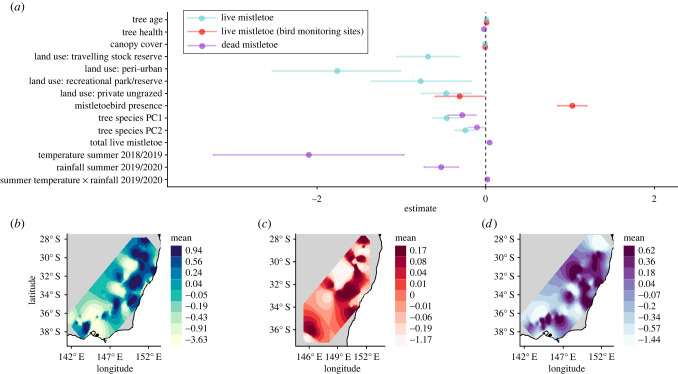
(*a*) Fixed effect estimates of the associations between environmental, biotic and climatic effects and mistletoe abundance and mortality. Land use factorial effects are relative to land use: national park/nature reserve. Points denote the posterior means and the error bars denote the 95% credibility intervals for the effects. Only significant fixed effects or factor levels (where estimates ± 95% credibility intervals do not overlap zero) from the top model, based on lowest DIC, are shown. See electronic supplementary material, figure S7 for the full model. (*b*–*d*) Spatial fields for the SPDE random effect of response variables of live mistletoe abundance (*b*), live mistletoe abundance including bird data (*c*), and dead mistletoe abundance (*d*), based on habitat (*b*,*d*) or bird (*c*) monitoring point locations (figure 1). Predictions are derived using the *ggField* function from the PointPolygon package v. 0.1.0 [47]. Table 1 and electronic supplementary material, file S1 for further information on the fixed effects and factor levels. (Online version in colour.)

Models including data from all habitat monitoring sites found mistletoe abundance was positively associated with the proportion of trees present with a DBH exceeding 80 cm, but negatively associated with canopy cover (figure 2*a*). Relative to within national parks and nature reserves, mistletoe abundance was lower in areas where land uses were predominantly private and ungrazed, travelling stock reserves, recreational parks/reserves or peri-urban. Mistletoe abundance was negatively associated with both the tree species composition measures (principal components 1 and 2), suggesting a higher abundance in plant community types dominated by white box *Euclyptus albens,* yellow box *E. melliodora*, river she-oak *Casuarina cunninghamiana* and mugga ironbark *E. sideroxylon,* relative to communities dominated by swamp mahogany *E. robusta*, paperbarks *Melalauca* spp., smoothed barked apple *Angophora costata* and other gum species (electronic supplementary material, figure S3). Supplementary analyses reinforced such relationships to the individual mistletoe and host tree species level (electronic supplementary material, figure S6). Presence of mistletoebirds was the dominant explainer of high mistletoe abundance at sites in which bird surveys were conducted. Despite their despotic impact on small woodland birds, there was no negative effect of mean noisy miner abundance on mistletoe abundance (electronic supplementary material, figure S8).

Dead mistletoe abundance was associated negatively with summer temperatures in 2018/2019 and with summer rain in 2019/2020 (i.e. peak drought). There was also a weak positive interaction between summer rainfall and maximum temperatures on mistletoe mortality in 2019/2020 (figure 2*a*). The spatial term showed a latitudinal trend in dead mistletoe abundance, with higher mortality in northern regions of southeastern Australia (figure 2*c*). For all three response measures, the inclusion of the SPDE effect substantially improved model fit: live mistletoe ΔDIC (from models excluding SPDE term) = −556, conditional *R*^2^ = 0.74; live mistletoe (birds) = ΔDIC−66 *R*^2^ = 0.35; dead mistletoe = ΔDIC = −195, *R*^2^ = 0.79.

*Question 2: what is the relationship between live mistletoe abundance and woodland bird abundance, and how does this relationship change during drought?*

**Figure 3.**
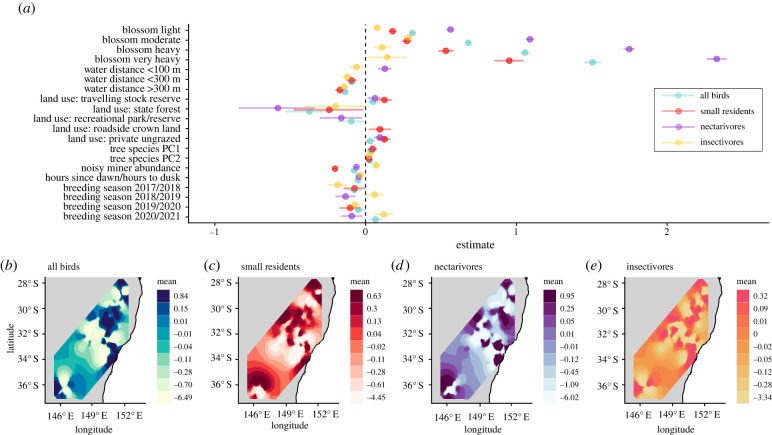
(*a*) Fixed effect estimates of the association between environmental, biotic and climatic effects and woodland bird abundance. Factorial effects are relative to the following levels: blossom = absent; water distance = 0 (i.e. water present); land use: national park/nature reserve; breeding season = 2016/2017. Points denote the posterior means and the error bars denote the 95% credibility intervals for the effects. Only significant fixed effects or effects with significant factor levels (where estimates ± 95% credibility intervals do not overlap zero) from the top models, based on lowest DIC, are shown. See electronic supplementary material, figure S8 for the full model summary; (*b*–*e*) spatial fields for the SPDE random effect of response variables of total (*b*), small resident (*c*), nectarivorous (*d*) and insectivorous (*e*) woodland bird abundance based on bird monitoring point locations (figure 1). (Online version in colour.)

Total live mistletoe abundance was not retained as a single term in the top model of overall bird abundance or any models of the bird functional sub-groups (figure 3*a*). Bird abundance was primarily driven by blossom abundance, with the greatest association with nectarivores and the weakest association with insectivores (figure 3*a*). There was substantial annual variation in bird abundance, primarily in nectarivores and insectivores. Noisy miner abundance had a negative association with overall bird abundance, primarily driven by impacts on small residents and nectarivores (figure 3*a*). Bird abundance was broadly, but weakly, positively associated with both tree species composition measures (tree species principal components 1 and 2). Effects of vegetation structure in the form of canopy and shrub cover extent were nominal. Relative to within national parks and nature reserves, overall bird abundance was lower in state forests and recreational parks/reserves and higher in travelling stock reserves and private ungrazed property. Bird abundance tended to decrease with increasing distance from a water source and time since dawn or dusk (figure 3*a*).

**Figure 4.**
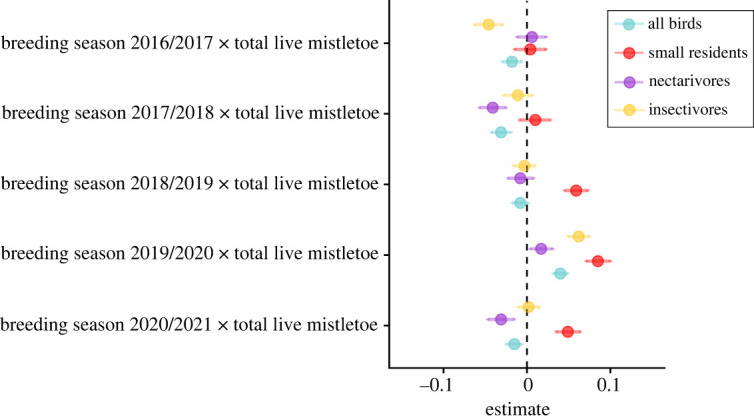
Fixed effect estimates of the interaction between breeding season × live mistletoe abundance and woodland bird abundance. Points denote the posterior means and the error bars denote the 95% credibility intervals for the effects. Estimates are derived from the same model as shown in figure 3 and electronic supplementary material, figure S8. (Online version in colour.)

The association between the abundance of remaining live mistletoes and woodland birds varied annually, and was most strongly positive during the peak drought breeding seasons of 2018/2019 and, in particular, 2019/2020 (figure 4). Associations with the abundance of live mistletoe increased most substantially during the drought for small residents and insectivores (figure 4). The association between both mistletoe and eucalypt blossom strengthened during the drought, particularly for nectarivores (mistletoe blossom) and insectivores (eucalypt blossom, electronic supplementary material, figure S9). Inclusion of the SPDE term again improved the fit of all four bird models: all birds ΔDIC (from models without SPDE term) = −2642, *R*^2^ = 0.71; small residents ΔDIC = −1228, *R*^2^ = 0.53; nectarivores ΔDIC = −3935, *R*^2^ = 0.87; insectivores ΔDIC = −1685, *R*^2^ = 0.40.

## 4. Discussion

Using a spatially extensive dataset spanning a 5-year period before, during and after a major drought, we show that the association between mistletoes and woodland birds strengthens during severe drought, and therefore that mistletoes may play a key role in moderating the negative impacts of below-average rainfall and above-average temperatures on vertebrate abundance. Paradoxically, we found substantial drought-associated mistletoe dieback in parts of southeastern Australia. Given the increased frequency and severity of drought and heatwave events predicted under impending climate change [19], our results suggest mistletoes are at risk from large-scale die-off over coming decades. Mistletoe die-off could have cascading impacts on community composition [4], with the greatest effects on resident and insectivorous species that are least able to avoid drought and heatwave effects via dispersal or dietary plasticity. We highlight the need for further research and long-term, spatially extensive monitoring to better understand the dynamics and drivers of mistletoe mortality and to inform conservation actions to maintain their keystone role in functioning ecosystems globally.

Land-use was a key predictor of live mistletoe abundance, with relatively lower abundances occurring in peri-urban areas such as street trees and recreational parks and reserves. We also found a strong positive relationship between mistletoe abundance and mistletoebird presence, a mistletoe specialist and the primary disperser of mistletoe seeds [15]. Mistletoebirds are scarce in suburban areas, although further work is needed to disentangle cause and effect in the relationship between mistletoebirds and mistletoe abundance.

We also found effects of tree species composition on mistletoe abundance. Many mistletoe species are specialists on a small range of host tree genera [39], with specialisation at the genus or family level associated with increased susceptibility to drought [10]. In terms of our study species, needle-leaf mistletoes are specialists on she-oaks, which fringe riparian zones throughout southeastern Australia. Box mistletoe tends to parasitize box–gum–ironbark tree species that predominate in more western woodlands within the study range [39], while long-flowered mistletoe depends primarily on hosts in the Myrtaceae spotted gum–ironbark forests in the north and east of the study range.

There was widespread and often high rates of mistletoe mortality throughout the study range, with needle-leaf mistletoe approaching 100% mortality in some core regent honeyeater breeding areas. Riparian corridors are critical drought refugia for many bird species [28] and in heavily cleared valleys [48], needle-leaf mistletoe nectar and fruit have become a primary breeding resource for multiple bird species [49]. Mass needle-leaf mistletoe mortality rapidly renders large stretches of core breeding habitat unviable for an entire assemblage of threatened species [50].

Mistletoe mortality was particularly high at lower latitudes towards the north of the study range, where drought effects were most pronounced. These spatial patterns support evidence from the INLA models that identified climatic predictors of mistletoe die-off. There were negative associations between dead mistletoe abundance and both maximum summer temperature and summer rainfall in the peak drought years of 2018 and 2019. Previous work describing drought-induced mortality in mistletoes documented marked differences between species, with 4% mortality in grey mistletoe *Amyema quandang* parasitizing Acacias, but 31% for harlequin mistletoe *Lysiana exocarpi* epiparasitic on the grey mistletoe. This difference is instructive—although both species were subjected to the same temperature regime, the epiparasitic species would have experienced more than double the water deficit given losses in the intermediate host [6]. Woodlands in the north of our study range were particularly badly affected by drought impacts, with hilltops and riparian corridors suffering widespread *Eucalyptus* and *Casuarina* mortality, respectively [25]. The results of our models reflected these trends, detecting a negative association between overall tree health and dead mistletoe abundance (figure 2*a* and electronic supplementary material, figure S7). The effect was small, however, suggesting that drought-induced die-off of host tree species only partly explains observed patterns of mistletoe mortality and that mistletoes are unlikely to be a key driver of host-tree mortality during drought [5,10].

Mistletoe mortality showed high positive spatial autocorrelation at the local scale of 0–6 km. Since rainfall and temperature data showed consistent annual differences throughout the study range, additional factors other than climate effects may be driving small-scale spatial structure in mistletoe die-off. High proportions of dead mistletoe could be explained by low recruitment due to the local extinction of key seed-dispersing animals such as mistletoebirds due to factors other than mistletoe die-off. Loss of seed dispersers may be stochastic in fragmented habitats [51], or due to the impact of despotic competitors such as noisy miners [44]. We found no evidence that mistletoe mortality was linked to mistletoebird absence, nor noisy miner abundance, but our models of live mistletoe abundance and woodland birds suggest that noisy miner presence (and mistletoebird absence) could be barriers to mistletoe recovery in areas in which they suffer mass mortality.

Mortality could also be driven by more nuanced local factors such as topography and geology. Aspect and height also modulate insolation and nutrient concentration; two factors known to affect mistletoe establishment and growth [1,3]. Regional-scale climatic forcing, coupled with the increasing proportion of remnant vegetation on rocky hillsides and other low productivity landforms diminishes the capacity of trees to host mistletoes to maturity [52], squeezing mistletoes to those few remaining catchment landforms where moisture and nutrient availability suffice. In addition to fundamental differences in water availability and cation concentrations, different host species exhibit divergent architectures, rates of evapotranspiration and physiological responses to acute heat and water deficit [10], subjecting mistletoes within their canopies to contrasting microclimates that may exacerbate the direct effects of climatic factors. Land management strategies could also cause mistletoe die-offs. Pesticides, livestock effluent, salinity changes and water abstraction may themselves, or through interactions with climate extremes, host vigour or animal associates push mistletoes beyond stress thresholds. Novel pathogenic infections may also kill mistletoes, but there is currently no evidence of pathogen-induced mistletoe die-off.

Importantly, none of the factors implicated in mistletoe mortality are region-specific, with many of the suspected drivers related to land-use intensification and climate change. Rather than being peculiar to eastern Australia, regional-scale mistletoe mortality may well be occurring in other parts of the world [5,13]. Although frequently overlooked by forest scientists, ecologists and restoration practitioners, our findings reinforce the emerging view that mistletoes are bioindicators of environmental health, challenging the preconception that these parasites necessarily kill their hosts and devalue wildlife habitats [3,53].

As a single term, our models did not identify mistletoe abundance as a key predictor of woodland bird abundance. By far the strongest predictor of woodland bird abundance was blossom abundance, which included both eucalyptus and mistletoe species in the blossom score. Blossom abundance was not only associated with high abundance of nectarivorous species but also of small residents and insectivores. This suggests that booms in eucalypt and mistletoe blossom have cascading trophic impacts [20] or that, through lagged responses to rainfall [54], blossom is a bioindicator of broader ecosystem productivity in space and time. Land use, water proximity, vegetation community and noisy miner abundance were the other main factors explaining woodland bird abundance. However, these effects have been researched extensively by others [44,55–57] but were important to control for in the modelling process rather than areas of interest *per se*.

There was a significant positive interaction between mistletoe abundance and breeding season on bird abundance in the peak drought breeding seasons of 2018/2019 and 2019/2020 (figure 4). These results suggest that mistletoes could play a key role in sustaining local bird populations during prolonged dry periods when other resources such as eucalypt blossom, invertebrates and seeds are limited. Associations with surviving mistletoes were strongest during the drought for small residents and insectivores. Many nectarivores are nomadic or migratory and can therefore avoid the worst impacts of temperature and rainfall extremes by undertaking long-distance movements to coastal refugia [58,59]. However, small residents, many of which are insectivorous, are limited in their ability to avoid drought impacts via dispersal [46]. Exploitation of microhabitat features such as live mistletoes could be the difference between life and death for small residents and insectivores during severe drought [46,60].

Because we accounted for mistletoe nectar in the blossom scores, the positive interaction between live mistletoe abundance and breeding season during the drought event suggests that some benefits of mistletoes for woodland birds during drought reach beyond the provision of nectar resources [45,46]. These may include nesting resources [9], or invertebrate availability, both within the canopy and on the forest floor. As with other parasitic plants, mistletoe-enriched litter boosts litter-dwelling invertebrate abundance, including those preferentially consumed by insectivorous birds [46,61]. Our results may also reflect an indirect association between mistletoe and birds during drought, such that other, unexplained factors driving bird abundance may also predict mistletoe abundance. We aimed to control for many of these potential factors, including distance to water, canopy and shrub cover as well as tree species composition. Clearly, more work is needed to identify the mechanisms underpinning the observed patterns.

Our study paints a worrying picture that as droughts become more frequent and severe in coming decades widespread mistletoe die-off is a very real risk. Mistletoe mortality is but one mechanism by which impending climate shifts could have cascading impacts at higher trophic levels. Now is the time to improve monitoring of mistletoe populations, particularly with broad-scale longitudinal data that until now may not have been considered necessary, to better understand the dynamics and drivers of their mortality, address threats through conservation actions and thus minimize the impacts of the decline of mistletoes from ecosystems globally.

## Data Availability

All bird and habitat data and associated R code are available via the Dryad Digital Repository at https://doi.org/10.5061/dryad.76hdr7sxp [62]. Due to the sensitive nature of the data involving Critically Endangered species we have offset the spatial location data. Electronic supplementary material is available online [63].
